# Enhancing oral English learning through AI: a case study on the impact of AI-driven speaking applications among Chinese university students

**DOI:** 10.3389/fpsyg.2025.1595818

**Published:** 2025-12-01

**Authors:** Xiudi Zhang

**Affiliations:** School of Foreign Languages, Shandong Technology and Business University, Yantai, China

**Keywords:** oral English, AI, Chinese university students, apps, case study

## Abstract

**Introduction:**

The integration of artificial intelligence (AI) into oral English learning has emerged as a promising solution to challenges such as limited practice opportunities, delayed feedback, and the demand for personalized learning experiences. However, empirical evidence on its effectiveness in university English education remains limited.

**Methods:**

This study adopted a qualitative case study design to examine the impact of AI-driven English-speaking applications on Chinese university students’ oral proficiency. Six students participated in semi-structured interviews after a 16-week semester, during which they completed structured after-class AI-supported speaking practices and reflective tasks using applications such as Liulishuo.

**Results:**

Findings indicate that AI applications effectively personalize learning by adapting practice content to individual proficiency levels and providing instant, data-driven feedback. Students reported noticeable improvements in pronunciation, grammar, and fluency, as well as increased motivation and engagement due to interactive features and gamified feedback mechanisms.

**Discussion:**

Despite these benefits, AI remains limited in replicating the emotional, cultural, and contextual nuances of human communication. Therefore, a blended model that integrates AI tools with traditional teacher-led instruction is recommended. The study offers practical implications for educators and developers seeking to optimize oral English learning through AI-enhanced pedagogical design.

## Introduction

In recent years, artificial intelligence (AI) has made significant strides in various fields, including education. In particular, the integration of AI into English language learning, especially oral English skills, has garnered attention. AI provides a conducive learning environment for learning English. Depending on their present level of English and their career requirements, it can significantly develop a customized environment where students employ their senses to simultaneously practice their English language abilities. AI improves practical abilities like writing and offers a realistic simulation dialogue platform like spoken English. It maximizes the teaching impact of English in ELT and expands students’ practice capacity. Advances in technology and platforms have made learning English easier. The study of how to use big data to enhance the effectiveness of oral English instruction is still in its early stages, though (Liu, 2024). Through a thorough literature review and empirical research, this study seeks to close this research gap and investigate and evaluate how big data-based oral English teaching strategies might increase teaching effectiveness. To clearly guide the direction of this study, the key research questions addressed are as follows:

1. How do AI-driven English-speaking applications impact the oral English proficiency of Chinese university students?
2. What are the key benefits and challenges faced by students when using AI applications for English learning?
3. How do AI tools support the development of personalized learning experiences in oral English education?

## Literature review

One of the key AI technologies in oral English education is speech recognition. AI-driven speech recognition systems, such as Google’s speech-to-text or Apple’s Siri, allow learners to practice pronunciation and fluency. Research shows that AI can analyze pronunciation accuracy, fluency, and speech rate in real time, providing instant feedback, such as pronunciation corrections and vocabulary suggestions (Ran et al., 2021). For example, AI speech recognition models can detect pronunciation errors and offer corrective suggestions, helping learners improve their pronunciation accuracy. The value of this approach is further underscored from an academic perspective. According to research by Evers and Chen (2022) these systems offer immediate feedback on pronunciation accuracy, helping learners identify mispronounced words and improve their speech. These technologies analyze the phonetic quality of spoken language and compare it with native speaker models, providing corrective suggestions. However, Liakina and Liakin (2023) pointed out that while speech recognition systems are useful for detecting mispronunciations, they may struggle with identifying subtleties in accents or certain regional variations in speech, limiting their effectiveness for a diverse group of learners.

### Natural language processing (NLP) in conversational AI

Another prominent AI application in oral English learning is conversational AI powered by natural language processing (NLP). AI chatbots provide learners with immersive oral practice by simulating real-life conversation scenarios. Research indicates that AI chatbots can deliver personalized dialogue practice, instant feedback, and language support, thereby enhancing learners’ oral fluency and self-confidence (Junaidi, 2020). Chatbots like Duolingo, Rosetta Stone, and HelloTalk use NLP algorithms to engage learners in interactive conversations (Czébely, 2023). These AI systems simulate dialogues with learners, providing real-time responses to their spoken input. According to Ram et al. (2018), conversational AI can help learners practice real-life communication skills in a risk-free environment. NLP models analyze the grammatical structure and context of learners’ speech, providing contextualized feedback. This interaction encourages spontaneous speech production and fosters a sense of communicative competence. Moreover, these platforms also leverage AI to adapt to the learner’s proficiency level. A study by Strielkowski et al. (2024) highlighted how adaptive learning systems powered by AI adjust conversation complexity based on the learner’s progress. This personalized approach increases learner engagement and enhances learning outcomes by providing an optimal challenge level.

### Pronunciation and accent training

AI tools have also been widely adopted to help learners improve their pronunciation and reduce accent interference. Speech synthesis technologies, such as Text-to-Speech (TTS), are employed to model native-like pronunciation (Saadia, 2023). As evidenced by the work of Gawate (2019), TTS systems are used to provide learners with examples of proper pronunciation, which they can emulate. In addition, AI algorithms can analyze the rhythm, intonation, and stress patterns in learners’ speech and compare them to native speaker patterns. This feature is particularly beneficial for learners who do not have consistent access to native speakers for oral practice.

### Personalized learning path

The personalization of language learning is increasingly driven by data-driven approaches. By analyzing a learner’s progress and performance, AI systems can tailor curricular content and exercises, thereby optimizing the development of vocabulary and pronunciation (AbdAlgane and Jabir Othman, 2023). Furthermore, sophisticated recommendation engines leverage this data to propose learning materials and activities that align with the individual’s specific proficiency and interests (Almaiah et al., 2022). While data-driven approaches offer the prospect of personalization, their reductive approach to language learning’s complexities, potential to limit perspectives, and inherent risk of algorithmic bias demand a cautious and critical perspective on their so-called “optimizing” role.

To some extent, AI technologies hold significant promise for enhancing oral English learning. As these technologies continue to evolve, they are expected to play an increasingly important role in shaping the future of language education. However, only a small number of academics have examined how to correct learners’ spoken language errors, and their research has shown that artificial intelligence (AI) may successfully enhance learners’ spoken language and positively impact learners’ spoken language correction (Rejeb et al., 2024). Few of the studies that look into spoken language correction use college students as their subjects, and the majority focus on the technology known as automatic speech recognition (ASR)(Li, 2020; Liu et al., 2024). In contrast, English-speaking applications (APPs) not only use speech recognition technology but also offer a variety of services that help college students speak more fluently and practice voice-overs, among other things. Research on these APPs is currently quite limited.

A similar reserach on improving EFL speaking performance among undergraduate students with an AI-powered mobile was condtuced by Mingyan et al. (2025), while effective for controlling variables and establishing causality, treats the actual process of AI-assisted learning as a “black box.” We are informed that features like “process-oriented monitoring” and “tailored instructions” were used, yet how these functions were understood and utilized by students in specific contexts remains unclear. Were students interacting with the AI proactively or merely completing tasks passively? Did they feel motivated or frustrated upon receiving automated feedback? How did the technological tool (Liulishuo) and the social tool (WeChat) interact within their learning ecosystem? Qualitative research, by collecting learning logs, conducting in-depth interviews, and observations, can open this black box, mapping out the complex, dynamic, and emotionally charged journey of technology integration into language learning.

Therefore, this study explores the impact of English-speaking applications (APPs)on Chinese university students. The necessity of conducting research on university students’ experiences with AI lies in the profound implications AI technologies have on educational paradigms, cognitive development, and future workforce readiness. As AI becomes increasingly integrated into academic environments, understanding how students interact with, perceive, and utilize these tools is crucial for optimizing educational outcomes. This research can elucidate the benefits and challenges students face, such as enhanced learning efficiency versus potential over-reliance on AI. Moreover, it can provide insights into the development of critical thinking and problem-solving skills in an AI-augmented context. By examining these experiences, educators and policymakers can design more effective curricula and support systems that harness AI’s potential while mitigating its risks, ultimately fostering a more informed and adaptable generation of graduates.

## Theoretical framework

The Interaction Hypothesis, proposed by Long (1983), suggests that language learners can significantly enhance their linguistic abilities through interaction with others, particularly in real communicative contexts. In this theoretical framework, interaction serves not only as a source of language input but also as a central element in the language learning process (Chen and Xu, 2023). Through interaction, learners receive abundant language input while simultaneously correcting errors through feedback, thereby promoting the improvement of their language skills.

In the development of speaking skills, the Interaction Hypothesis emphasizes that continuous interaction with others -such as teachers, peers, or intelligent systems-enables learners to improve their fluency and accuracy in real-world contexts through feedback (Zheng and Yu, 2020). Feedback not only helps learners identify deficiencies in their language use but also stimulates their ability to self-correct, creating a positive language learning cycle. Research shows that the error correction process in interaction is crucial for the enhancement of speaking abilities, particularly as AI technologies allow learners to frequently correct errors in a low-pressure environment, providing numerous opportunities for language practice in a short period.

In modern language learning contexts, especially through interaction with AI, learners can continuously receive instant feedback, correct errors, and deepen their understanding of language rules through repeated practice (Fitria, 2021). This interactive learning approach not only improves the accuracy of language expression but also boosts learners’ confidence in using the language. Through ongoing interaction and feedback, learners’ speaking abilities are significantly enhanced, providing a more solid foundation for their language application.

In this study, which explores the use of AI for improving English speaking skills among Chinese students, the Interaction Hypothesis provides a foundational theoretical framework (Li and Wang, 2021). The hypothesis posits that language acquisition is optimized when learners engage in meaningful communication and receive feedback that helps correct errors and refine language use. In the context of AI-assisted language learning, this theory is particularly relevant as AI systems offer interactive platforms where students can practice speaking and receive real-time corrections.

Given that many Chinese students face challenges in oral English proficiency, largely due to limited opportunities for spontaneous communication in authentic contexts, AI provides a unique solution (Zhai and Wibowo, 2023). By engaging with AI, students can simulate real-world interactions, receiving immediate corrective feedback without the social pressures of traditional classroom settings (Zhang and Zhang, 2023). This continuous interaction, paired with feedback, aligns with the principles of the Interaction Hypothesis, which emphasizes that language learners improve not only through exposure to language input but also through the active process of error correction and adjustment based on feedback.

Furthermore, AI systems allow for individualized learning experiences, where students can engage in repeated practice, correct mistakes, and gradually build confidence in their speaking abilities. This iterative process is crucial for the development of oral proficiency, as it fosters an environment where students are consistently refining their language skills through interaction and feedback. Therefore, the Interaction Hypothesis provides a compelling rationale for using AI as a tool to enhance oral English skills, especially in settings where traditional opportunities for interaction may be limited.

## Methodology

To gain deeper, more inclusive, and comprehensive insights into the university students’ experiences with AI, this study used a qualitative case study approach. The research employed naturalistic methods that mirrored how students interact with, perceive, and utilize these AI tools, engaging with participants in a way that was natural and unobtrusive (Lincoln and Guba, 1985; Rallis and Rossman, 2012).

Case studies are commonly used in qualitative research. Abercrombie et al. (1984) argue that while a case study cannot provide broadly applicable information, it is valuable in the early stages of research because it generates hypotheses that can be further tested with larger samples (p. 34). However, it is misleading to view the case study merely as a preliminary tool for surveys, as Flyvbjerg (2001) notes, because it offers context-dependent insights and supports epistemic and theoretical development.

Additionally, case study research is not about sampling from a larger population, such as selecting participants or organizations. The primary goal of the researcher is to thoroughly understand the case, whether through analytical, holistic, cultural, or mixed methods approaches—focusing, at least initially, on the case itself (Stake, 2005). As such, this research emphasizes the specific lessons that can be learned from studying Chinese university students’ experiencing of using AI tools in oral English learning — a unique moment in time and space.

### The study context

“Oral English” course is a compulsory course for the Primary Education program at Z University, designed to develop students’ essential professional competencies for English teaching. Through systematic training, the course helps pre-service teachers enhance the accuracy of their English language tone, master essential classroom English expressions, and conduct lessons fluently in English. By completing this course, students will become familiar with the organization of English and bilingual classroom teaching, acquire commonly used functional sentence patterns for teacher-student interaction, and effectively use English in everyday teaching activities, thereby significantly improving their practical classroom language skills.

A total of 44 s-year students enrolled in the course participated in the AI-supported learning activities. The duration of the study was 16 weeks ——one academic semester. AI applications were not directly used during classroom teaching, but rather assigned as after-class learning tasks to extend students’ oral practice beyond limited class hours. Specifically, students were required to:

1. Select an AI-driven oral English application of their own choice (e.g., Liulishuo, Qupeiyin, Doubao, etc.).
2. Complete one AI-supported oral practice task per week throughout the 16 weeks.
3. Write reflective notes after each practice, documenting their experiences, perceived benefits, and challenges when using the AI tool.

This approach allowed students to flexibly choose the AI applications most suited to their needs while ensuring consistency in practice frequency. By embedding AI practice as structured assignments, the study captured how AI tools functioned as supplementary aids in authentic learning contexts.

### Participant selection

The selection of participants for this study was conducted through a purposive sampling strategy to ensure the inclusion of students who are involved in the course of Oral English. At the end of the semester, students were invited to participate in follow-up interviews. Recruitment was initiated by disseminating an invitation via university mailing lists, online student forums, and departmental announcements. The invitation outlined the study’s objectives, the voluntary nature of participation, and the confidentiality of responses. Interested students were directed to complete a brief screening survey to assess their eligibility based on predefined criteria, such as current enrollment status, prior use of AI technologies, and willingness to participate in a semi-structured interview. Eligible participants were then contacted to schedule interviews at their convenience. To encourage participation, students were informed of the study’s potential impact on improving AI integration in education and were offered a small incentive, such as a gift card, as a token of appreciation for their time and contribution. This approach ensured a representative sample while maintaining ethical standards in participant recruitment.

From the pool of 44 students, six participants were recruited using purposive sampling, based on their willingness and availability to share in-depth reflections. Semi-structured interviews were then conducted face-to-face, each lasting 35–45 min. The interviews explored students’ usage patterns, emotional responses, perceived effectiveness, and challenges when using AI for oral English learning. The demographic features of the students are shown in Table 1.

**Table 1:** Interviewee demographics (n = 6).

Pseudonym	Gender	Interview data
Lucy	Female	5-12-2024
John	Male	7-12-2024
David	Male	13-12-2024
Adam	Male	21–12-2024
Mercy	Female	26-12-2024
Sam	Male	28-12-2024

The interviews followed a semi-structured format, beginning with pre-designed questions such as, “How frequently do you use AI applications (e.g., ChatGPT, Grammarly, or AI-based research tools) in your English study?,” “How do you feel about the increasing use of AI in education? Do you see it as beneficial, concerning, or both?,”and “How has your perspective on AI evolved since you first started using it?.” As the participants shared their experiences and reflections, additional spontaneous follow-up questions were asked to gather detailed accounts of their stories, perspectives, and emotions (Creswell, 2021).

This study relies on a small sample of six participants, which limits the generalizability of the findings. While the sample size is small, it is important to note that this research adopts a qualitative case study approach, which focuses on providing deep, context-specific insights into the experiences of a select group of Chinese university students using AI-driven English-speaking applications. The aim of this study is not to generalize the results to a broader population, but rather to explore the nuances of individual student interactions with AI tools in a controlled, educational environment.

In summary, while the small sample size limits the generalizability of the findings, the study offers valuable qualitative insights into the role of AI in enhancing oral English learning for university students, providing a foundation for further exploration in this area.

### The legitimacy of the study

The validity of this study was not derived from the sheer volume of data collected but rather from the carefully analyzed themes generated from the experiences and insights of individual participants. This analytical approach was neither purely deductive nor inductive; instead, it followed a hypothetic method (Creswell, 2021). The study aimed to gather and analyze the experiences of Chinese university students of using AI tools in oral English learning. This systematic process of collecting and theorizing key data is referred to as a systematic combination (Fairbrother, 2013). Through this process, the cognitive patterns of the participants were synthesized into overarching themes, ensuring coherence and consistency across the data (Stenhouse, 1977). Ultimately, these themes were developed to shed light on analyzing students’ experiences, educators can refine AI tools to better align with pedagogical goals, fostering more effective and equitable language learning environments.

### Ethical considerations

Given that this study required students to share their perspectives on experiencing in using APPs in study, careful attention was paid to ethical considerations. To ensure participants felt at ease, the interviews were conducted in a relaxed setting at the dining hall, with snacks and drinks provided to create a comfortable and welcoming atmosphere for the students.

### Text encoding

In this study, we employed reflexive thematic analysis, a method celebrated for its accessibility and theoretical flexibility in interpreting qualitative data. This methodology facilitates the systematic identification and analysis of patterns or themes within datasets (Braun et al., 2023). A fundamental principle guiding our research was the dedication to accurately representing students’ opinions and experiences, while also recognizing and addressing the reflexive impact of our own interpretations as researchers. Initially, we imported the interview data into NVivo software, where the material was analyzed and coded to create free nodes, which were subsequently organized. Secondary coding of these free nodes was conducted in alignment with the research objectives and direction.

**Figure 1:**
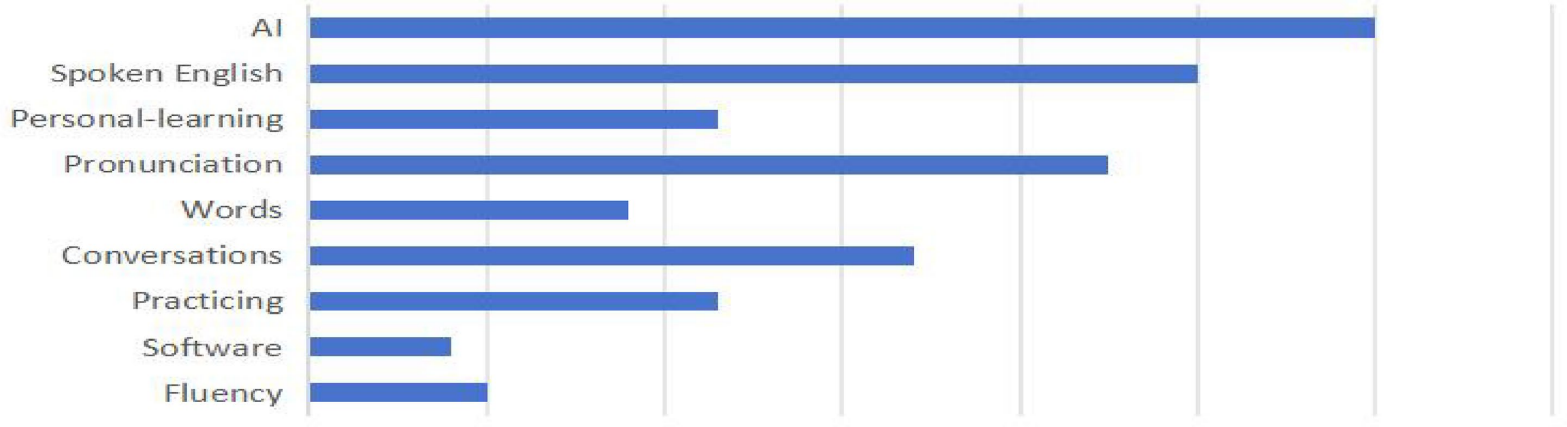
The percentage of the nodes.

Based on a synthesis of the interview outline, the research team categorized all coding variables into two groups: external factors and internal factors. The study ensures rigor and credibility by systematically coding and analyzing interview transcripts. The analytical steps are as follows:

1. Familiarization with data: The interview transcripts were repeatedly read and initially organized, with irrelevant expressions and fillers removed.
2. Initial coding: The cleaned interview data were coded in NVivo. Codes related to the research focus were labeled. A total of 54 free nodes, ranging from “N1-1” to “N46-5,” were generated.
3. Generating sub-themes: Each sentence was coded to produce initial codes, which were then grouped by similarity to form sub-themes. Through this process, nine sub-themes such as “AI,” “Spoken English,” and “Personal-learning” were generated. After the research team finalized the coding results, Figure 1 was produced.
4. Generating themes: Based on the research framework, the sub-themes and initial codes were clustered into broader themes. For example, “AI,” “Conversations,” “Practicing,” and “Software” were categorized under the theme External Factors.
5. Defining and reviewing themes/reliability testing: After clarifying the core meanings, the final thematic coding framework was established. Coding reliability was tested using Cohen’s Kappa coefficient, as it provides a more rigorous measure of agreement than the percentage agreement method by accounting for chance consistency. Two independent coders coded the same dataset, and NVivo’s “Coding Comparison Query” was used to extract and compare node data. The computed Kappa value was 0.800, indicating excellent inter-coder reliability according to standard benchmarks (Kappa > 0.75; Table 2).

**Table 2:** Results of the kappa test.

	Numbers	Asymptotic standard error	Approximate Tb	Asymptotic significance
Measure of agreement kappa	0.800	0.070	18.873	0.000
Valid cases	32			

The results indicate that factors influencing students’ English learning fall into two major categories: external factors and internal factors. External factors include the use of AI technology, simulated conversations, and language learning software, which enhance convenience, engagement, and practice opportunities. Internal factors relate to learners’ individual abilities and learning approaches, such as attention to fluency, pronunciation, and vocabulary acquisition, as well as personalized learning based on interests and goals. Students adjust their learning focus based on software feedback, improving their sense of rhythm and expressive ability through imitation and repetition, while reinforcement mechanisms consolidate learning outcomes. Overall, learners combine external resources with internal motivation and self-regulation, forming two interdependent dimensions of English learning (Appendix).

### Analysis

#### Personalized learning plans improve learning efficiency

One of the significant advantages of using AI learning tools for English language acquisition is their ability to create personalized learning plans tailored to individual proficiency levels and goals. This personalization allows students to receive a more targeted and efficient learning experience compared to traditional, one-size-fits-all classroom methods (Hurskaya, 2023). For instance, applications like Liulishuo offer a diagnostic test upon first use, which assesses the learner’s current level of English proficiency.

*Lucy: The learning software I use is Liulishuo. When I first enter the app, it asks me about my current English level and what level I’d like to reach after studying. I selected CET-4, pronunciation, and daily communication. It then creates a detailed plan for you. It also has a check-in function where you can set how long you want to study each day. I set it to study every day for 10 min. During my time using Liulishuo, I mainly worked on my English pronunciation, particularly reading words and sentences. After I finish reading, it gives me a score for my pronunciation. I feel that this not only helps me correct my pronunciation but also reinforces words I’ve learned and introduces new ones I have not encountered before. Liulishuo also has a dubbing feature, which I find very interesting—it feels like learning through play. There are also videos related to learning English, which have helped me understand more about the language. After some time studying with Liulishuo, I feel that my English pronunciation has become more standard, my vocabulary has expanded, and my listening skills have improved. I really find Liulishuo useful.*

*John: The AI tool I use is “Liulishuo.” When I first enter the app, it creates a personalized learning plan through its AI language learning features. Based on my English proficiency and learning goals, it tailors daily study tasks for me, covering vocabulary, grammar, listening, speaking, and reading. I can also freely arrange my specific weekly study schedule. Liulishuo uses images and example sentences to help me remember words. For example, with the word “ambitious,” it shows a picture of a determined person striving to achieve their goals, along with the sentence “He is an ambitious young man who wants to achieve great success.” This approach to word recognition has made a strong impression on me, and the app also schedules reviews based on the forgetting curve, ensuring that I truly master each word.*

According to Lucy and John, we can see that AI could able to design a customized curriculum that focuses on the student’s specific needs—be it vocabulary, grammar, listening, speaking, or pronunciation. This approach ensures that learners are not overwhelmed with content they have already mastered, while also providing adequate challenges to help them progress. Moreover, these tools adapt as the learner’s abilities improve, adjusting the complexity of exercises and offering new tasks that align with their evolving needs. This dynamic adaptation, coupled with daily learning tasks, helps students stay on track and motivated, as they can clearly see their progress and improvements over time.

#### Instant feedback and error correction enhance speaking skills

The integration of instant feedback and error correction in language learning has been widely recognized as a pivotal factor in enhancing speaking skills. Immediate feedback allows learners to recognize and rectify errors in real-time, fostering a more dynamic and responsive learning environment (Maier and Klotz, 2022). Instant feedback mechanisms, such as digital tools or teacher-led corrections, provide learners with the opportunity to adjust their speech output immediately, thereby reducing the likelihood of fossilization of errors.

Students generally believe that AI’s speech recognition and scoring systems help them correct pronunciation and grammar mistakes. For instance, some students found that Liulishuo accurately pinpointed pronunciation errors and provided repeated practice opportunities. Additionally, AI offers feedback on linking sounds, intonation, and stress, making their pronunciation more natural and accurate.

*Mercy: Since I made the decision to improve my English speaking skills, AI has become my personal language coach. I was quickly drawn to its engaging nature. Initially, I spent half an hour each day conversing with the AI, ranging from simple greetings to complex scenario simulations. It always provided immediate feedback, correcting my pronunciation and grammar mistakes.*

Mercy highlights the role of AI as a “personal language coach,” emphasizing its accessibility and adaptability to her learning needs. This aligns with contemporary research in educational technology, which underscores the potential of AI-driven tools to provide personalized and scalable learning experiences. At the end, Mercy also mentioned the error correction function by AI, the same as David experience as following:

*David: Every day, I would select classic English articles, movie dialogues, or news clips to practice shadowing. The AI would accurately recognize my pronunciation and compare it with the original audio, promptly pointing out any errors in my pronunciation or inaccuracies in my intonation. I would repeat the exercises until my pronunciation and intonation matched the original audio as closely as possible.*

David combined with AI’s ability to accurately identify and compare pronunciation deviations from native speaker models, creates a robust framework for error detection and correction. This aligns with the principles of corrective feedback in second language acquisition (SLA), where timely and specific feedback is crucial for learners to notice and rectify errors, thereby preventing fossilization (Albelihi and Al-Ahdal, 2024). The iterative process of repeating exercises until achieving close alignment with the original audio underscores the importance of repetition and reinforcement in consolidating correct linguistic patterns.

In conclusion, the combination of instant feedback and error correction serves as a powerful tool in the development of speaking skills. By creating an interactive and supportive learning environment, educators can empower learners to refine their oral proficiency and achieve greater communicative competence.

#### Interactivity and fun increase motivation to learn

AI technology enhances the interactivity and fun of English speaking learning, effectively increasing the motivation of Chinese university students and promoting the development of their speaking skills. AI-driven learning platforms can enhance learner immersion through continuous interaction with students (Xu, 2024). Compared to traditional passive learning models, AI provides a more interactive learning experience. In interaction with AI systems, students can continuously practice their speaking skills, which strengthens the persistence and motivation to learn.

Many students reported that AI learning software’s interactive features increased their interest in learning English. For example, dubbing exercises (such as in QupeiYin), situational dialogues (such as in Liulishuo), and role-playing (such as in Doubao) helped them improve their English while having fun. Some students used AI for simulated conversations in various real-life scenarios, such as at the airport or in a restaurant, which made learning more engaging and immersive.

*Sam: There are also methods that involve learning English through movies and TV shows. I watched “The Legend of the White Snake” and followed along with the video, which had English subtitles. I could dub over my favorite scenes, and I really enjoy this approach. It allows me to practice my English pronunciation while having fun, which makes the learning process both enjoyable and effective.*

*Lucy: Liulishuo also offers many fun learning modes, such as challenge games and check-in challenges. These modes add both fun and a sense of challenge to the learning process, allowing me to make continuous progress in a relaxed and enjoyable environment. At the same time, I can interact and compete with other learners, which further boosts my motivation to learn.*

#### AI as a learning aid but not a complete substitute for traditional learning

Although AI provides many conveniences in speaking practice, some students believe it still has significant limitations. For instance, some students found AI conversations to be somewhat rigid, lacking the natural flow, spontaneity, and emotional nuances of real human communication. This rigidity can make learning feel monotonous and less engaging over time. Additionally, while AI excels at correcting pronunciation and offering instant feedback, it often falls short in providing deeper linguistic insights (Lara et al., 2024). For example, AI may struggle to explain complex grammar rules, cultural context, or the subtle nuances of language usage that are crucial for achieving fluency.

*John: I started by practicing word repetition, but after 2 days, I found it rather uninspiring and monotonous. Later, I discovered scenario-based practice, where I could simulate speaking in different situations. While this was more interesting, my English proficiency made it difficult for me to practice fluently. I often had to pause and look up words, which disrupted the flow of the exercise.*

John mentioned that while initial word repetition practice helped with familiarizing pronunciation, it quickly became monotonous and lacked motivational appeal. This suggests that AI tools, when providing basic phonetic training, may rely too heavily on repetitive tasks without sufficiently incorporating context or stimulating learner interest. From an academic perspective, a single-mode language input approach (such as mechanical repetition) may fail to meet the cognitive demands of language learning, as language acquisition requires not only phonetic imitation but also semantic understanding, contextual association, and creative output (Swain, 1993). Therefore, when designing speaking exercises, AI tools should integrate more interactive and contextualized content to enhance learner engagement and improve learning outcomes.

Moreover, AI lacks the ability to adapt to the unique learning styles and individual needs of students in the way a human teacher can (Bajaj and Sharma, 2018). It cannot offer personalized encouragement, motivation, or mentorship, which are often key factors in a student’s progress. Real-life conversations and classroom interactions, on the other hand, provide opportunities for students to practice improvisation, interpret body language, and engage in meaningful cultural exchanges—elements that are difficult to replicate with AI.

*Adam: "When practicing speaking with AI, I found that while it can correct my pronunciation, it always feels like something is missing. For example, when I encounter grammar issues, AI can only provide simple corrections and cannot explain in detail why a certain usage is correct or how to apply it flexibly in different contexts, as a teacher would. Additionally, AI’s feedback is always mechanical and lacks emotional support. Sometimes, I wish I could receive some encouragement or specific advice tailored to my learning progress, but AI does not seem capable of doing that.”*

*“In contrast, I find interacting with teachers and classmates in the classroom much more helpful. For instance, teachers give personalized suggestions based on my performance and even design exercises based on my interests. In group discussions, I can not only practice spontaneous expression but also better understand others by observing their facial expressions and body language. These are experiences that AI cannot provide.”*

## Discussion

The findings of this study underscore the significant role that AI-supported tools play in enhancing oral English learning, particularly through personalized and interactive mechanisms. These outcomes align with prior research emphasizing the efficacy of AI in providing adaptive learning pathways and immediate feedback, which cater to individual learner needs and promote autonomous progress (Zhang, 2024). The students’ positive experiences with AI-driven platforms such as Liulishuo and movie-based imitation exercises reflect a broader trend in educational technology toward gamification and immersive learning, which have been shown to boost engagement and motivation.

The interview highlights two methods of promoting English learning through entertainment and interactive mechanisms, emphasizing the key role of fun and challenge in enhancing learning motivation. Sam mentions learning English through movies and TV shows, specifically watching *The Legend of the White Snake* with English subtitles and dubbing over his favorite scenes. This method not only helped him improve his English pronunciation but also made the learning process more enjoyable and effective (Shadiev et al., 2025). It integrates cultural elements with language learning, emphasizing the effectiveness of immersive learning and imitation in strengthening language skills. Research shows that immersive learning enhances learner engagement, particularly in pronunciation and oral expression, helping learners improve language fluency and accuracy in a relaxed environment (Yudintseva, 2023).

Lucy refers to the Liulishuo platform, which incorporates challenging games and check-in tasks, further enhancing the interactivity and fun of the learning process. These modes not only make learning more enjoyable but also stimulate continuous learning motivation through task challenges and a sense of accomplishment. The integration of interaction and competition allows learners to share progress with others, adding a social learning element. Research indicates that gamified learning, by providing instant feedback, reward systems, and competitive challenges, not only increases learner engagement but also strengthens their interest and commitment to learning (Alsawaier, 2018).

Overall, the experiences shared by both interviewees reflect that learning methods combining entertainment elements, interactive feedback, and challenging tasks not only improve language proficiency but also effectively stimulate learning motivation. Through such approaches, learners can enhance their language skills while enjoying the process, fully embodying the educational philosophy of “learning through fun.”

While prior studies have highlighted the efficiency and personalization of AI in language learning (Kovalenko and Baranivska, 2024; Kaswan et al., 2024; Hussain et al., 2024), this study adds nuance by showing that students also perceive risks of monotony and over-reliance on technology. Unlike human interlocutors, AI lacks the capacity to provide affective support and culturally grounded explanations. These divergences suggest that AI aligns with theoretical perspectives that emphasize interaction, output, and mediation, but it cannot fully embody the socio-affective dimensions of language learning.

From the findings, we can also see that many students believe that AI should be used as a supplementary tool rather than a complete replacement for traditional learning methods. Combining AI with classroom instruction, real-life conversations, and human feedback creates a more holistic learning experience. This blended approach allows students to benefit from the efficiency and accessibility of AI while still gaining the depth, adaptability, and richness of human interaction (Mulenga and Shilongo, 2025). Ultimately, AI serves as a powerful aid in language learning, but it cannot fully replicate the dynamic and multifaceted nature of traditional learning environments.

The findings of this study can also be critically examined through the lens of Interaction Hypothesis, revealing both significant alignments and notable points of departure. Long’s (1983) Interaction Hypothesis emphasizes the centrality of interaction and feedback in language development. The students’ experiences with instant feedback and iterative correction directly reflect this mechanism: by receiving immediate input on errors, learners engaged in a cycle of modification and self-correction that mirrors the negotiation of meaning in authentic interaction. Although AI-mediated communication differs from human dialogue, the findings show that AI tools can successfully simulate interactive feedback processes, thereby supporting oral proficiency (Fathi et al., 2024). Similarly, Swain’s (1993) Output Hypothesis is supported by students’ reports of being “pushed” to produce language through AI-speaking tasks, leading to heightened metalinguistic awareness and self-correction. From a Vygotskian perspective, AI functions as a mediational tool that scaffolds learning within the ZPD, though it falls short in facilitating the co-construction of meaning that characterizes human dialogue.

## Conclusion

The integration of artificial intelligence (AI) into oral English learning has demonstrated significant potential in addressing key challenges faced by learners, such as limited opportunities for practice, lack of immediate feedback, and the absence of personalized learning experiences. This study explored the impact of AI-driven tools, particularly English-speaking applications (APPs), on Chinese university students, revealing several critical insights into the benefits and limitations of AI in language education.

Firstly, AI tools such as Liulishuo and other similar platforms have proven effective in providing personalized learning plans tailored to individual proficiency levels and goals. By offering diagnostic tests and adaptive learning paths, these tools ensure that students receive targeted instruction, enhancing their learning efficiency and motivation. The ability of AI to adjust the complexity of exercises based on the learner’s progress fosters a more engaging and effective learning experience compared to traditional, one-size-fits-all approaches.

Secondly, the instant feedback and error correction mechanisms embedded in AI applications play a pivotal role in improving speaking skills. By providing real-time corrections on pronunciation, grammar, and intonation, AI tools help learners identify and rectify errors immediately, preventing the fossilization of mistakes. This feature is particularly beneficial for students who lack access to native speakers or consistent feedback from human instructors.

Thirdly, the interactivity and gamification elements of AI platforms significantly increase learners’ motivation. Features such as dubbing exercises, situational dialogues, and role-playing scenarios make the learning process more enjoyable and immersive. These interactive modes not only enhance engagement but also encourage continuous practice, which is essential for developing fluency and confidence in spoken English.

Theoretically, these findings resonate with and extend established frameworks in second language acquisition. Consistent with Long’s Interaction Hypothesis, students’ experiences demonstrated that iterative interaction with AI feedback supported oral proficiency development. From the lens of Sociocultural Theory, AI tools functioned as mediational artifacts that scaffolded learners’ progress, while the lack of authentic human collaboration highlighted their limitations. Taken together, the findings show that AI applications align with core principles of Second Language Acquisition, but they also expose boundaries where human mediation remains irreplaceable.

However, the study also highlights the limitations of AI in replicating the nuanced and dynamic nature of human communication. While AI excels at providing structured feedback and repetitive practice, it often falls short in delivering the emotional and cultural depth that human interactions offer. The rigidity of AI conversations and the lack of adaptability to individual learning styles underscore the importance of combining AI tools with traditional classroom instruction and real-life interactions.

In conclusion, AI serves as a powerful supplementary tool in oral English learning, offering personalized, efficient, and interactive learning experiences. However, it should not be viewed as a complete substitute for traditional teaching methods. A blended approach that integrates AI with human instruction and real-world practice is essential for creating a holistic and effective language learning environment. As AI technologies continue to evolve, further research is needed to explore their long-term impact on language acquisition and to develop strategies for maximizing their potential in educational settings. This study contributes to the growing body of literature on AI in education, providing valuable insights for educators, policymakers, and developers aiming to enhance language learning outcomes through innovative technologies.

## Data Availability

The raw data supporting the conclusions of this article will be made available by the author, without undue reservation.
