# Supplementary material for: Enhancing oral English learning through AI: a case study on the impact of AI-driven speaking applications among Chinese university students
Source: Front Psychol. 2025 Dec 1;16:1595818. doi: 10.3389/fpsyg.2025.1595818 (PMC12706583; doi:10.3389/fpsyg.2025.1595818)
Supplement: Supplementary file 1 [file Table_1.docx]

Appendix

Thematic Analysis: Examples of Thematic Coding Results

| Theme | Sub-theme | Example Excerpts from Raw Data |
| --- | --- | --- |
| External Factors | AI | Overall, this software has been greatly helpful for me. I used to be reluctant to practice spoken English, thinking it was troublesome and unnecessary. Now with AI, I find oral practice much easier and more convenient. I believe that with persistence, I will make gradual progress. (N4-6) |
|  | Conversations | AI can simulate a variety of real-life dialogue scenarios. It plays different roles in the conversation—such as teacher and student, or waiter and customer—making me feel as if I were in an authentic communicative environment. (N11-2) |
|  | Practicing | The software I use is Liulishuo (Speaking English Fluently). It allows users to dub classic works and provides pronunciation scoring after each attempt. I can retry multiple times, and after each round, I can listen to the original version to identify my weaknesses. (N13-1) |
|  | Software | Recently, I have been using a dubbing app called Fun Dubbing. I personally find it very interesting. The app offers many shows, animations, and films I like, including English dramas and Hollywood movie clips. By imitating their speech and tone, I can improve my spoken English. (N20-1) |
| Internal Factors | Fluency | After practice, the system provides scores for accuracy, fluency, and completeness. This helps me clearly identify which aspects I need to improve. (N36-1) |
|  | Personal-learning | The Liulishuo app has provided me with a completely new learning experience. Its content is rich and diverse—covering daily English, business English, and exam preparation. This allows me to study according to my goals and interests, greatly enhancing my motivation. (N37-1) |
|  | Pronunciation | I began using the AI companion Doubot in college, mainly for English speaking practice. When teachers assigned us to imitate CET-4 or CET-6 listening sentences, I would use the AI app to read along, imitate pronunciation and intonation, and improve my sense of rhythm and fluency. (N10-1) |
|  | Spoken English | Overall, learning spoken English through AI was refreshing for me. It provided a real conversational environment where I could correct my pronunciation and express myself more naturally. I hope AI can play an even greater role in future English learning. (N46-5) |
|  | Words | I mainly focus on pronunciation practice—reading words and sentences. After reading, the app scores my pronunciation. This not only helps me correct errors but also reinforces vocabulary I already know while introducing new words. (N1-1) |

The frequency analysis further reveals that within “External Factors,” the sub-themes **“Conversations”** and **“AI”** appeared most frequently, indicating the central role of AI-based interaction tools in current English learning practices. Within “Internal Factors,” **“Spoken English,” “Personal-learning,”** and **“Pronunciation”** were the most frequently mentioned, reflecting students’ growing focus on communicative competence and personalized engagement.
